# Two decades of Ghana’s National Health Insurance Scheme: perceived service quality and membership renewal among informal sector workers

**DOI:** 10.1080/16549716.2026.2755137

**Published:** 2026-11-03

**Authors:** Abdul-Rahman Bunyamin, Seohyun Lee

**Affiliations:** a Department of Public Administration, Yonsei University Graduate School Mirae Campus, Wonju, Republic of Korea; b Department of Nursing, Technical University College, Tamale, Ghana (TUCG); c Department of Global Public Administration, Yonsei University Mirae Campus, Wonju, Republic of Korea; d Department of Global Health, University of Washington, Seattle, WA, USA

**Keywords:** Health service quality, national health insurance scheme, informal sector, Ghana, health insurance membership

## Abstract

**Background:**

Two decades into Ghana’s National Health Insurance Scheme (NHIS), concerns about service quality and drop-out persist.

**Objectives:**

This study aimed to measure multidimensional perceived service quality and its associations with NHIS membership renewal among informal-sector workers in two socio-economically distinct regions in Ghana.

**Methods:**

Building upon the classic frameworks on service quality, a cross-sectional survey was conducted. Descriptive comparison followed by multivariable logistic regressions was performed, adjusting for sociodemographic characteristics selected based on conceptual relevance and the significance of unadjusted estimates.

**Results:**

Four hundred and eight informal sector workers, including street vendors, artisans, and farmers, participated in the study. Among them, 265 (65.0%) were active NHIS subscribers, 108 (26.5%) were drop-outs, and 35 (8.6%) were never enrolled. The study found that service quality perceptions differed significantly (*p* < 0.05) by insurance status, with NHIS dropouts reporting the least favorable experiences across all quality dimensions. The regression estimates showed that respondents who obtained all or some of their prescribed medications or diagnostic tests had higher odds of renewing their membership than those who did not. Reasonable waiting times for consultation (aOR = 5.86, CI: 3.27–10.49) and for diagnostics (aOR = 7.57, CI: 3.61–15.88) were positively associated with renewal. Professional staff disposition (aOR = 3.60, CI: 2.05–6.34) and a better doctor–patient relationship (aOR = 2.79, Cl: 1.90–4.11) were also significantly associated with higher odds of NHIS renewal.

**Conclusions:**

This study underscores the need for effective resource allocation to improve service quality across critical dimensions. Findings can inform health policy in similar settings, reflecting perceived service quality as key to health insurance retention.

## Paper Context

- **Main findings**: Amid a low NHIS renewal rate, our findings suggest that multidimensional perceived service quality is associated with NHIS renewal among informal sector workers in Ghana.
- **Added knowledge**: Building upon the classic frameworks on service quality, we contextualized the multidimensional nature of perceived health service quality from the client perspective and demonstrated its association with membership renewal among informal sector workers in two socioeconomically contrasting regions of Ghana.
- **Global health impact for policy and action**: Public health insurance schemes in low-resourced settings should prioritize service quality as a strategic policy instrument for advancing universal health coverage in line with the Sustainable Development Goal Target 3.8.

## Background

The delivery of health services costs money, and ultimately someone has to pay [1]. Decisions about who pays, payment arrangements, and the burden they place on individuals are crucial issues that shape healthcare access and utilization. Consequently, healthcare financing has long been a major concern for policymakers globally [2]. Particularly, in sub-Saharan Africa, where the majority are low- and middle-income countries (LMICs), budgetary constraints have significantly challenged healthcare delivery [3,4]. Recognizing this hurdle, a strong case has been made for health insurance as a key policy instrument that provides financial risk protection [5,6] and promotes utilization across various healthcare services [7–9].

Health insurance programs may come in different forms and with distinct operational arrangements [10]. However, they generally converge on a common purpose of creating the necessary conditions for subscribers to share the risk of sickness by transferring unexpected healthcare costs into fixed premiums [2]. In essence, subscribers pay premiums in exchange for the insurer’s commitment to cover unforeseen health expenditures and deliver quality health services. Following this principle, sub-Saharan African countries have experimented with health insurance programs as part of reform initiatives to increase access to health services [11,12]. In this regard, Nigeria, Ghana, Kenya, Uganda, and Tanzania are a few examples that have implemented various health insurance programs [13].

Among these countries, Ghana has made significant healthcare financing reforms through the enactment of the Health Insurance Act (Act 650) in 2003, which led to the National Health Insurance Scheme (NHIS) implemented in 2004. In the context of Ghana, the NHIS is a public health financing program that aims to offer quality health services to all residents, regardless of age, gender, ethnicity, or nationality [14]. The program’s objectives are to promote access to high-quality healthcare and advance social health protection through risk equalization, solidarity, and equity [15]. Essentially, the NHIS has been considered one of Ghana’s most remarkable social intervention programs [16].

Despite its successful implementation, the scheme encounters significant setbacks [13]. First, it is well established that enrollment figures are much lower than anticipated, with the disproportionately low representation of disadvantaged populations [17]. In its 2018 Annual Report, the National Health Insurance Authority (NHIA) estimated that the enrollment as a percentage of the national population was 39%, 41%, and 39% as of 2014, 2015, and 2016, respectively [18]. The same report pegged enrollment in 2017 at 37% and 36% in 2018. Furthermore, in the first quarter of 2020, active membership was estimated at only about 12 million, which was approximately 40% of Ghana’s population [19]. For a program intended to promote universal health coverage and lessen disparities in access to quality health services, these enrollment figures can be too low to achieve its intended purposes [20]. Similar health insurance schemes elsewhere in sub-Saharan Africa have likewise struggled with low enrollment. For instance, the public health insurance programs in Kenya, Nigeria, and Tanzania are fragmented in character, with much lower enrollment rates [21].

Related to this phenomenon is potentially the most crucial setback of Ghana’s NHIS, where the growing number of existing subscribers drop out of the scheme. Estimates show that 41% of existing members in 2014 dropped out in 2015, and 53% of the active members in 2015 also failed to renew in 2016 [22]. Other scholars with similar research interests have also reported high drop-out rates in Ghana and in other parts of Africa [4,19,23]. As highlighted by Nsiah-Boateng et al., substantial discontinuation of NHIS enrollment can negatively impact financial risk protection, particularly for the vulnerable segments of the scheme, thereby hindering efforts to achieve universal health coverage [22].

Against this backdrop, previous studies have examined factors associated with NHIS membership decisions. While studies have reported various determinants of current enrollment status, limited attention has been paid to the renewal decisions among existing members [24]. Moreover, economic and socio-demographic characteristics constitute the commonly used set of predictors, without adequately addressing the complexity of cognitive and behavioral motivation [17,24,25]. Consequently, the empirical literature presents significant gaps in understanding the dynamics behind membership retention.

In fact, the quality of health services under NHIS has been questioned over the years and frequently cited as a major disincentive for health insurance membership [14,16,26]. Notwithstanding subsidized premiums and exemptions for vulnerable populations, sub-Saharan African countries still struggle with low enrollment rates and notably poor service quality as a potential cause [27]. As one of the most significant factors for health insurance enrollment and retention, health service quality has been widely discussed in previous literature. However, little attention has been given to the subscribers’ perceptions of service quality grounded in firsthand experience in the context of Ghana’s NHIS membership renewal decisions. A mapping study highlights this gap, emphasizing how Ghana’s efforts in improving health service quality have largely remained provider-oriented, with limited integration of communities’ or service users’ perspectives [28]. Moreover, previous studies that included a health service quality component in the empirical models generally overlook its multi-dimensionality. Although a few studies analyzed multidimensional aspects of subscribers’ perceptions, they do not adequately capture the recent empirical evidence among NHIS subscribers [29]. By revisiting the classic frameworks on service quality, this study aimed to address the multi-dimensionality of perceived service quality.

More importantly, there are significant variations in enrollment among diverse population segments, with those in the informal sector disproportionately less represented in Ghana’s NHIS. Despite being the largest segment of Ghana’s labor force, as highlighted in a previous study, the informal sector population constituted about 32% of active NHIS membership in 2018 [18,30]. Also, of the 12 million active NHIS members in the first quarter of 2020, only 34% were from the informal sector, while all formal sector employees with automatic deductions from their social security contributions are exempt from yearly premium payment [19]. This makes the informal sector a unique population segment suitable for a study that seeks to understand the relationship between perceived service quality and NHIS membership renewal.

Focusing on health service quality under NHIS in Ghana, this study therefore investigated how perceptions of multidimensional health service quality are associated with the membership renewal among informal sector workers. In doing so, the perceptions of three service quality dimensions, namely patient flow, resource availability, and relational quality, were examined. The study hypothesized that better perceived service quality across the three dimensions will have a positive effect on the continuation of membership.

## Methods

### Study design, setting, and sampling

A cross-sectional survey was conducted with respondents in the informal sector as the only population segment that pays the yearly NHIS premium in Ghana [17,31]. The study sites were purposively selected in the capital cities of Greater Accra Region (Accra) and Northern Region (Tamale) to reflect the geographic and economic divides of the country. Identified among the poorest regions in Ghana, the Northern Region was targeted alongside the most urbanized and economically vibrant Greater Accra Region. In fact, a previous study conducted in Greater Accra and Western Regions highlighted the need for including the Northern part of the country in the analysis to provide a more comprehensive landscape of socioeconomic context and health insurance coverage in Ghana [32].

As an initial step in recruitment, two communities were randomly chosen each from Accra and Tamale. The survey respondents were subsequently recruited within these four communities using convenience sampling due to practical challenges in compiling a comprehensive list of informal sector workers for simple random sampling. The respondents were approached for voluntary participation if they were aged between 18 and 69 and belonged to the informal sector. All premium-exempt groups were excluded from the study. To recruit eligible participants in community settings, trained research assistants visited households, workshops, and street vendors. Efforts were made to recruit broader informal sector categories in general, such as street vendors, artisans, commercial vehicle operators, farmers, laborers, and traders. However, no systematic quota or stratification by occupation type was employed during recruitment. Similar to other studies, if multiple individuals were eligible and willing to participate from a single household or workshop, only the first two available respondents were allowed to participate [33].

The survey data were collected electronically via Google Forms using a mixed-mode approach comprising both self- and interviewer-administered questionnaires. Of the 408 responses included in the final analysis, 342 (83.8%) completed the questionnaire with the assistance of trained enumerators who entered participants’ responses directly into Google Forms. The remaining 66 respondents (16.2%) completed a self-administered online questionnaire independently and were asked to complete the questionnaire only once to minimize the risk of duplicate responses. Responses appeared broadly similar across the two survey modes. The sample size was set at 400, with a sampling error of ±4.9% point at 95% confidence level. Although the online survey link was expected to be manually closed after 400th respondents, a buffer of 10 responses in the sample was allowed during the data collection process. A sample size was calculated based on assumptions for logistic regression using the R WebPower package. The NHIS retention rate, disaggregated by perceived service quality, was not available a priori, so the overall retention probability of 67.7% reported in a study from Ghana was used as a conservative proxy for the reference group (those with low perceived service quality). The minimum sample size of 359 was required to detect an odds ratio of 2.0 for those with high perceived service quality (80% power, α=0.05) [34]. The odds ratio of 2.0 was assumed as a conventional effect size for behavioral studies without available empirical estimates.

### Measurements

The outcome variable is NHIS membership status, and the key independent variable is the perception of multidimensional service quality at NHIS-approved health facilities that a respondent visited the last time. For many decades, measuring quality of care has been a major concern in health services research literature, and the conceptualization of quality has evolved over time. Among different approaches to measuring health service quality, two seminal works of Donabedian and Parasuraman et al. have been investigated as cornerstones. Donabedian’s framework sets out the systems-based model to assess quality by capturing structure, process, and outcome [35]. In defining quality of care, Campbell et al. revisited Donabedian’s model and claimed that the quality of care is essentially defined as whether individuals can access the structure and process of care, and additionally whether it is effective in terms of clinical and interpersonal care [36]. According to this definition, access and effectiveness adequately explain the concept of quality.

Another widely used conceptual model in public health research, with marketing as its origin, is the SERVQUAL by Parasuraman et al. The model summarizes the dimensions of perceived quality into tangibles, reliability, empathy, assurance, and responsiveness [37]. In a systematic review of dimensions of service quality in healthcare, Fatima et al. found that SERVQUAL was the most frequently adopted framework among 214 included studies [38]. Particularly, some contextual dimensions such as perceived waiting time or adequacy of resources were added in studies from developing countries [38].

Incorporating these discussions from the previous literature, this study aimed to measure service quality from the perspective of NHIS subscribers. Access to structure and process was measured in terms of resource availability and patient flow, while the effectiveness of interpersonal care was measured as relational quality. Although the effectiveness of clinical care is another important part of health service quality measurement, it was not included in the measurement due to practical challenges related to its provider-oriented technical nature [39]. Furthermore, this study attempted to capture the NHIS subscribers’ experiences in service quality measurement rather than the clinical aspects examined by providers. The specific measurement scale was developed by contextualizing the Ghanaian NHIS and the existing literature on public health service quality measurement [40]. The conceptual illustration of service quality dimensions employed in this study is presented in Figure 1.

**Figure 1.**
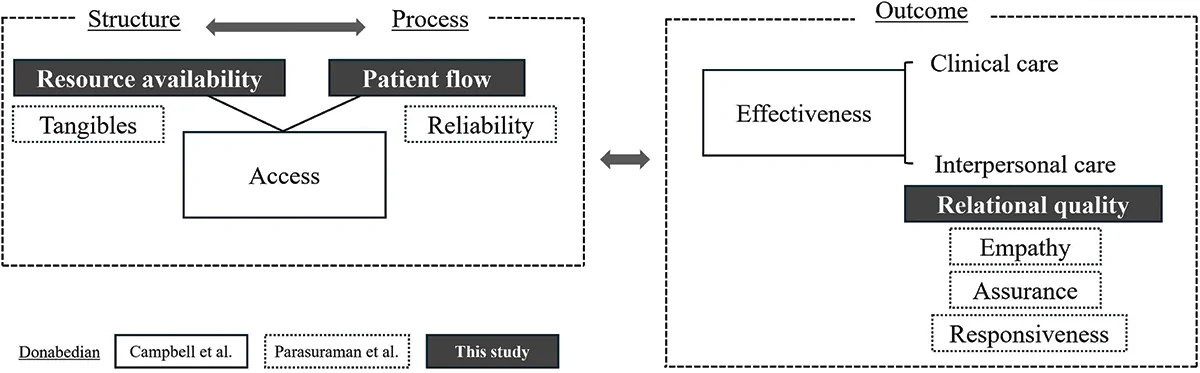
Adapted conceptual framework of public health service quality measurement under NHIS in Ghana [35–37].

### Data collection and analysis

The data were collected between April and July 2023 from respondents with different NHIS membership statuses. In accordance with the terms of IRB approval, the consent process was conducted entirely within the Google platform, rather than through a traditional written form. Respondents were given information about the study, their rights and confidentiality before ticking the consent checkbox electronically. The study was conducted following the principles of the Declaration of Helsinki.

The structured questionnaire mainly asked questions on health insurance statuses and respondents’ perceptions of health service quality based on their recent experience. Key study population characteristics were also collected. The questionnaire is available in Supplementary File 1. The questionnaire incorporated skip patterns, in which selected service quality items were administered only to respondents for whom the questions were applicable. In the regression model, these conditional variables and other missing values were excluded from the analysis through listwise deletion, which is a default procedure in Stata (version 18, StataCorp LLC) [41].

The adapted conceptual framework presented in Figure 1 was used to develop the dimensions of service quality measured in this study. To assess the dimension of resource availability based on prior literature, the respondents were first asked whether they were prescribed any medications or recommended for any diagnostic tests [20,25,40,42–44]. If they answered yes, then, they were asked to choose one of the three responses for the availability of both medications and diagnostic tests: all, at least some, or none of the medications or diagnostic services were available at the NHIS-accredited health facility they last visited. The responses were dichotomized into ‘all or at least some were available’ and ‘none was available’ for analysis.

Similarly, the measurement of patient flow dimension was informed by previous studies that highlighted waiting time as a key component of perceived service quality [40,43–46]. The respondents rated their perceived waiting time for consultations and diagnostic test, if applicable, on a 5-point Likert scale ranging from ‘not long at all’ to ‘very long’. The responses for consultations and diagnostic tests were dichotomized into ‘long (very long, long)’ and ‘not long (acceptable, not long, and not long at all)’.

Guided by existing literature, the measurement of relational quality dimension focused on the professionalism of administrative staff and the relationship with a healthcare provider [40,42,47–49]. The respondents were asked if the staff disposition was professional or not on a 5-point Likert scale, ranging from ‘not professional’ to ‘very professional’. The responses were dichotomized into ‘not professional (not professional at all, not professional)’ and ‘professional (somewhat professional, professional, very professional)’. Further, the doctor–patient relationship was measured by averaging responses on a four-item, 5-point Likert scale anchored by ‘strongly disagree’ and ‘strongly agree’. The four question items address the sufficiency of time spent with a physician, the comprehensiveness of the examination, the clarity of explanations, and the sufficiency of information to improve health. The measurements for demographic characteristics were also regrouped into binary variables for more intrinsic interpretation of the results.

The NHIS membership status was categorized into four groups: renewed from previous enrollment, dropped out, never enrolled, and newly enrolled. A total of 410 respondents participated in the survey, of which two newly enrolled respondents were excluded from subsequent analysis as the response category was too small to permit meaningful statistical comparison or to yield reliable estimates. Descriptive statistics were used to summarize the respondents’ characteristics, NHIS membership status, and multidimensional perceived service quality among participants who renewed from previous enrollment, dropped out, and never enrolled. A chi-square test and ANOVA were performed as an initial step to examine the associations between perceived service quality and NHIS membership status.

Multivariable logistic regression analysis was conducted for each service quality dimension, adjusting for study sample characteristics that showed statistical significance in the unadjusted analyses. Gender, age, marital status, educational attainment and region of respondent satisfied the screening threshold (*p* < 0.05) and were therefore included as covariates. On the other hand, variables such as income, religion and self-reported health status were excluded based on the threshold. However, the residence variable, despite being significant, was intuitively excluded as well because it serves a similar function to the region variable, which was included for Ghana’s geographic distinctions and their associated socioeconomic gaps into the sample. Respondents who had never enrolled were excluded from the regression analysis, considering their lack of experience with service quality under NHIS. Diagnostic checks were performed prior to model estimation to test the key assumptions of logistic regression analysis. The multicollinearity among the predictors was assessed using Variance Inflation Factors (VIFs), while linearity between the continuous predictor (doctor–patient relationship) and the logit of the outcome was examined using the Box–Tidwell test. The results (see Supplementary File 2) satisfied these key conditions of logistic regression as used in this study.

## Results

### Characteristics of study sample

Of the 408 respondents included in the analysis, 265 (65.0%) were active NHIS subscribers who renewed their previous membership, while 108 (26.5%) respondents had dropped out. Another 35 (8.6%) respondents had never enrolled but were included for descriptive comparison to examine the relationships between NHIS membership status and multidimensional perceived service quality. Figure 2 illustrates the study sample based on the NHIS membership status.

**Figure 2.**
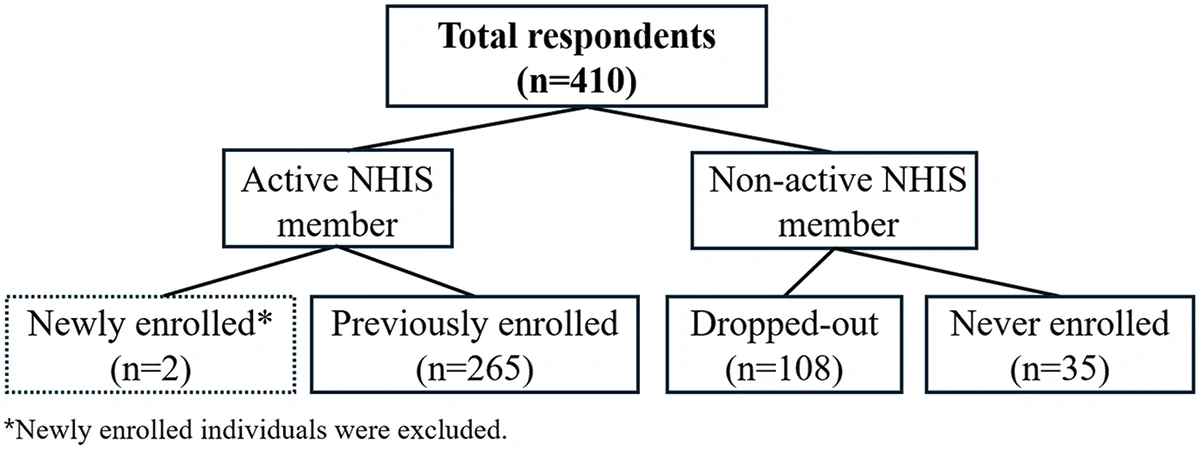
Sample characteristics according to the NHIS membership status.

Table 1 shows the sociodemographic characteristics of the respondents. Approximately half of the respondents were female (53.7%), married (53.4%), and from the Northern Region (52.5%). Most of them had at least basic education (78.7%) with 57.8% residing in urban areas. Less than 50% had a monthly income of 1,000 Ghc (USD 64) or more (43.6%), and artisans accounted for the highest proportion (36.0%) among occupation categories. Respondents aged between 18 and 29 (37.7%) and 60–69 (4.7%) constituted the highest and the least represented age categories, respectively.

**Table 1.** Characteristics of study sample **(*N* = 408).**

	n (%)		n (%)
NHIS Status		Marital Status	
Renewed from previous membership	265 (65.0%)	Single	179 (43.9%)
Dropped-out	108 (26.5%)	Married	218 (53.4%)
Never Enrolled	35 (8.6%)	Widowed/divorced	11 (2.7%)
Gender		Region	
Male	189 (46.3%)	Northern	214 (52.5%)
Female	219 (53.7%)	Greater Accra	194 (47.5%)
Education Level		Residence	
No formal education	87 (21.3%)	Peri-urban*	172 (42.2%)
At least basic education	321 (78.7%)	Urban	236 (57.8%)
Approximate Monthly Income [Ghc]		Age	
Less than 100 (appx. USD 6)	6 (1.5%)	18–29	154 (37.7%)
100–399 (appx. USD 6–25)	26 (6.4%)	30–39	79 (19.4%)
400–699 (appx. USD 25–45)	86 (21.1%)	40–49	96 (23.5%)
700–999 (appx. USD 45–64)	112 (27.5%)	50–59	60 (14.7%)
1000 (appx. USD 64) or above	178 (43.6%)	60–69	19 (4.7%)
Primary Occupation		Self-Reported Health	
Food/street vendor	69 (16.9%)	Very poor	1 (0.2%)
Artisan	147 (36.0%)	Poor	33 (8.1%)
Laborer	25 (6.1%)	Fair	85 (20.8%)
Trader/business person	99 (24.3%)	Good	137 (33.6%)
Commercial motor/vehicle driver	35 (8.6%)	Very good	152 (37.3%)
Farmer	24 (5.9%)	Religion	
Others	9 (2.2%)	Muslim	216 (52.9%)
		Christian	192 (47.1%)

*Original response category of ‘rural’ was recoded as ‘peri-urban’ due to its transitional features between urban and rural settings.

### Reasons for drop-out and perceived health service quality by NHIS membership status

Among 108 respondents who dropped out of the scheme, it was found that different reasons accounted for membership drop-out, with poor service quality being the most cited, as showed in Figure 3. The results show that 39.8% regarded poor service quality as the main reason for membership discontinuance, followed by no confidence in the scheme (36.1%).

**Figure 3.**
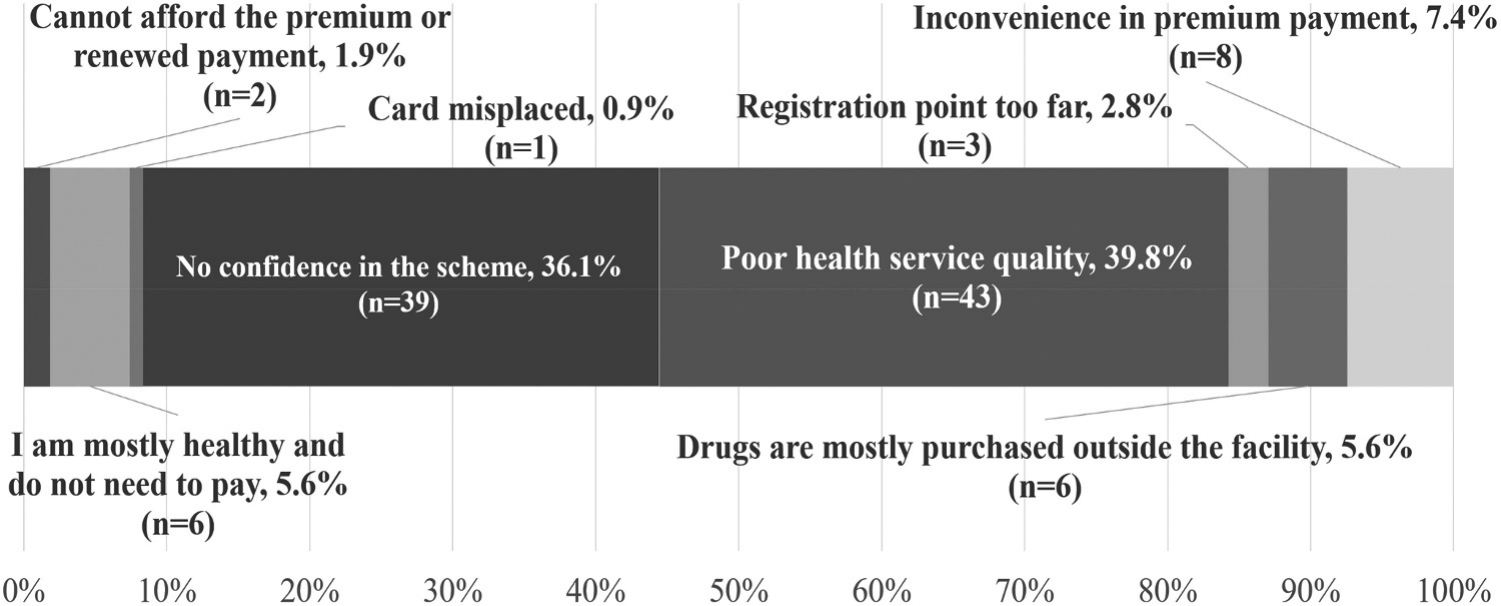
Reasons for drop-out (*n* = 108).

The results from Table 2 show that the respondents’ experiences in terms of resource availability, patient flow, and relational quality were statistically significantly different across health insurance statuses. Specifically, those who dropped out of NHIS reported the least favorable experience, with the highest percentages of medication (33.3%) and diagnostic test (41.7%) unavailability. They also showed the highest percentages of experiencing long waiting times for consultation (69.4%) and diagnostics (69.4%). The dropout group reported their experience as notably less positive than the renewed or never enrolled groups, with the lowest percentage perceiving staff disposition to be professional (36.0%). The dropout group also had the lowest mean score for doctor–patient relationship among the three insurance groups (Mean 3.30; SD 0.91).

**Table 2.** Perceived service quality by NHIS membership status.

Dimension of Perceived Health Service Quality:		NHIS Membership Status		
		n (%)		
Resource Availability		Renewed	Dropped out	Never enrolled
Medications (χ^2^, *p* < 0.001)		(*n* = 265)	(*n* = 99)	(*n* = 35)
	None was available	16 (6.0%)	33 (33.3%)	1 (2.9%)
	All or at least some were available	249 (94.0%)	66 (66.7%)	34 (97.1%)
Diagnostics (χ^2^, *p* < 0.001)		(*n* = 249)	(*n* = 96)	(*n* = 34)
	None was available	17 (6.8%)	40 (41.7%)	0 (0%)
	All or at least some were available	232 (93.2%)	56 (58.3%)	34 (100%)
Patient Flow		Renewed	Dropped out	Never enrolled
Waiting time for consultation (χ^2^, *p < 0.001)*	Long	(*n* = 265)	(*n* = 98)	(*n* = 35)
		61 (23.0%)	68 (69.4%)	6 (17.1%)
	Not long	204 (77.0%)	30 (30.6%)	29 (82.9%)
Waiting time for diagnostics (χ^2^, *p < 0.001)*		(*n* = 244)	(*n* = 62)	(*n* = 34)
	Long	67 (27.5%)	43 (69.4%)	1 (2.9%)
	Not long	177 (72.5%)	19 (30.6%)	33 (97.1%)
Relational Quality		Renewed	Dropped out	Never enrolled
Staff disposition (χ^2^, *p < 0.001)*	Not professional	(*n* = 265)	(*n* = 100)	(*n* = 35)
		86 (32.5%)	64 (64.0%)	3 (8.6%)
	Professional	179 (67.5%)	36 (36.0%)	32 (91.4%)
		Mean (SD) (responses on a four-item, 5-point Likert scale)		
Doctor–patient relationship (ANOVA, *p* < 0.001)		(*n* = 265)	(*n* = 102)	(*n* = 35)
		3.99 (0.63)	3.30 (0.91)	4.6 (0.37)

### Association between perceived service quality and NHIS renewal

Table 3 presents the association between perceived resource availability and NHIS renewal decision among respondents with prior NHIS enrollment history (i.e. either renewed their NHIS enrollment or dropped out). The results from Models 1 and 2 indicate that resource availability, specifically the availability of medications (aOR = 5.74, 95% CI: 2.66, 12.42) and diagnostic tests (aOR = 10.33, 95% CI: 4.83–22.09), were significant factors associated with NHIS renewal decisions. Those who accessed all or at least some medications or diagnostic tests had significantly higher odds of NHIS renewal than those who got none. Except for region of respondents which was significant in only Model 2, the age, gender, marital status, and education were important demographic factors significantly associated with membership renewal in both Models 1 and 2, as shown in Table 3.

**Table 3.** The association between resource availability and NHIS renewal among individuals with prior enrollment history.

		Resource Availability Dimension	
Independent Variables	Unadjusted Estimates	Model 1: Availability of Medications	Model 2: Availability of Diagnostic Tests
	uOR [95% CI]	aOR [95% CI]	aOR [95% CI]
Gender			
Male	Ref	Ref	Ref
Female	3.89 [2.42–6.28]**	3.13 [1.78–5.53]**	2.96 [1.61–5.43]**
Age			
≤40	Ref	Ref	Ref
>40	5.1 [3.01–8.64]**	4.63 [2.52–8.51]**	4.95 [2.58–9.51]**
Marital Status			
Single	Ref	Ref	Ref
Married	3.38 [2.11–5.40]**	1.97 [1.12–3.46]*	2.49 [1.36–4.55]*
Education			
No Formal Edu.	Ref	Ref	Ref
At least Basic Edu.	2.96 [1.71–5.14]**	3.24 [1.63–6.43]*	4.43 [2.10–9.37]**
Region			
Northern	Ref	Ref	Ref
Greater Accra	1.96 [1.24–3.11]*	1.74 [0.99–3.07]	2.16 [1.17–4.01]*
Residence			
Peri-urban	Ref		
Urban	5.83 [3.58–9.50]**		
Approximate Monthly Income [Ghc]			
Less than 700	Ref		
700 and more	1.49 [0.92–2.39]		
Religion			
Islam	Ref		
Christianity	0.82 [0.52–1.28]		
Self-Reported Health			
Fair, good, or very good	Ref		
Poor or very poor	1.77 [0.70–4.45]		
Availability of Medication			
None was available	Ref	Ref	
All or some were available	7.78 [4.03–14.99]**	5.74 [2.66–12.42]**	
Availability of Diagnostics			
None were available	Ref		Ref
All or some were available	9.74 [5.15–18.45]**		10.33 [4.83–22.09]**

uOR: unadjusted odds ratio; aOR: adjusted odds ratio;*p < 0.05; **p < 0.001.

Furthermore, the patient flow dimension was also found to be significantly associated with NHIS renewal decisions. The results from Table 4 show that those who perceived the waiting time to consult a doctor as ‘not long’ had significantly higher odds of renewing their membership compared with those who reported it to be long (aOR = 5.86, 95% CI: 3.27, 10.49). Similarly, perceiving the waiting time to receive diagnostic services as ‘not long’ was associated with 7.57 times higher odds of membership renewal compared with those who perceived the waiting time to be long (aOR = 7.57, 95% CI: 3.61, 15.88). Some covariates were also significantly associated with membership continuance in Models 3 and 4. For instance, gender, age, and educational attainment were found to have a significant association with NHIS renewal in both models. Marital status was significant in Model 3 but lost significance in Model 4, whereas region was not significant in both models.

**Table 4.** The association between patient flow and NHIS renewal among individuals with prior enrollment history.

		Patient Flow Dimension	
Independent Variables	Unadjusted Estimates	Model 3: Waiting Time for Consultation	Model 4: Waiting time for Diagnostic Tests
	uOR [95% CI]	aOR [95% CI]	aOR [95% CI]
Gender			
Male	Ref	Ref	Ref
Female	3.89 [2.42–6.28]**	3.77 [2.08–6.82]**	4.58 [2.18–9.61]**
Age			
≤40	Ref	Ref	Ref
>40	5.1 [3.01–8.64]**	4.13 [2.22–7.69]**	6.12 [2.77–13.54]**
Marital Status			
Single	Ref	Ref	Ref
Married	3.38 [2.11–5.40]**	2.00 [1.12–3.58]*	1.96 [0.96–3.99]
Education			
No Formal Edu.	Ref	Ref	Ref
At least Basic Edu.	2.96 [1.71–5.14]**	2.65 [1.29–5.42]*	4.48 [1.96–10.23]**
Region			
Northern	Ref	Ref	Ref
Greater Accra	1.96 [1.24–3.11]*	1.67[0.93–2.99]	1.87 [0.91–3.86]
Residence			
Peri-urban	Ref		
Urban	5.83 [3.58–9.50]**		
Approximate Monthly Income [Ghc]			
Less than 700	Ref		
700 and more	1.49 [0.92–2.39]		
Religion			
Islam	Ref		
Christianity	0.82 [0.52–1.28]		
Self-Reported Health			
Fair, good, or very good	Ref		
Poor or very poor	1.77 [0.70–4.45]		
Waiting Time for Consultation			
Long		Ref	
Not long	7.58 [4.52–12.70]**	5.86 [3.27–10.49]**	
Waiting Time for Diagnostics			
Long			Ref
Not long	5.98 [3.25–10.98]**		7.57 [3.61–15.88]**

uOR: unadjusted odds ratio; aOR: adjusted odds ratio;*p < 0.05; **p < 0.001.

Again, the estimates from Table 5 indicate that relational quality had significant and positive associations with the decision to renew NHIS membership. Model 5, shows that the odds of membership renewal were 3.60 times higher among the respondents who experienced professional staff disposition at NHIS-accredited facilities, compared with those who reported unprofessional staff disposition (aOR = 3.60, 95% CI: 2.05, 6.34). Similarly, a unit improvement in the doctor–patient relationship scale was associated with 2.79 times higher odds of membership renewal (aOR = 2.79, 95% CI: 1.90, 4.11). With the exception of the region of respondents, which was not significant in Model 6, all covariates were significantly associated with membership renewal in the two models for relational quality (Models 5 and 6).

**Table 5.** The association between relational quality and NHIS renewal among individuals with prior enrollment history.

		Relational Quality Dimension	
Independent Variables	Unadjusted Estimates	Model 5: Staff Disposition	Model 6: Doctor–patient Relationship
	uOR [95% CI]	aOR [95% CI]	aOR [95% CI]
Gender			
Male	Ref	Ref	Ref
Female	3.89 [2.42–6.28]**	4.33 [2.43–7.70]**	3.76 [2.11–6.71]**
Age			
≤40	Ref	Ref	Ref
>40	5.1 [3.01–8.64]**	4.59 [2.51–8.42]**	3.90 [2.12–7.20]**
Marital Status			
Single	Ref	Ref	Ref
Married	3.38 [2.11–5.40]**	1.91[1.09–3.34]*	2.14[1.21–3.78]*
Education			
No Formal Edu.	Ref	Ref	Ref
At least Basic Edu.	2.96 [1.71–5.14]**	3.46 [1.73–6.92]**	3.54 [1.74–7.21]**
Region			
Northern	Ref	Ref	Ref
Greater Accra	1.96 [1.24–3.11]*	1.82 [1.04–3.20]*	1.49 [0.84–2.63]
Residence			
Peri-urban	Ref		
Urban	5.83 [3.58–9.50]**		
Approximate Monthly Income [Ghc]			
Less than 700	Ref		
700 and more	1.49 [0.92–2.39]		
Religion			
Islam	Ref		
Christianity	0.82 [0.52–1.28]		
Self-Reported Health			
Fair, good, or very good	Ref		
Poor or very poor	1.77 [0.70–4.45]		
Staff Disposition			
Unprofessional	Ref	Ref	
Professional	3.70 [2.28–6.00]**	3.60 [2.05–6.34]**	
Doctor–patient relationship			
None was available	3.25 [2.31–4.57]**		2.79 [1.90–4.11]**

uOR: unadjusted odds ratio; aOR: adjusted odds ratio;*p < 0.05; **p < 0.001.

## Discussion

By examining public health insurance subscription and retention, this study demonstrates that informal sector workers in Ghana tend to incorporate multiple dimensions of perceived service quality into their NHIS membership renewal decisions, beyond sociodemographic factors. Specifically, perceived service quality was measured across three dimensions: resource availability, patient flow, and relational quality. The findings suggest that positive perceptions of service quality along these three dimensions are key to the continuation of NHIS subscriptions. It can be inferred that perceived service quality has major implications for the retention and growth of NHIS membership, and hence the sustainability of the national health insurance program in Ghana.

To be specific, the availability of medications or diagnostic tests under the resource availability dimension stood out as a critical factor. The odds of existing subscribers renewing their membership increase if they were provided with all or at least some of the prescribed medications or diagnostic test when using NHIS services. Notably, resource constraints under Ghana’s NHIS are also documented in other studies. For instance, it has been reported that despite having insurance coverage, 63% of respondents paid out-of-pocket for medications, and 35% obtained drugs from outside the health facility [50]. Similar to these previous findings, this study also demonstrated that the time spent waiting for consultation or diagnostic test within the patient flow dimension is a key factor closely linked to decisions regarding NHIS membership renewal. Lastly, the quality of the interactions with health professionals or staff was identified as another important factor associated with the renewal decisions.

Overall, it is understood that improvement in perceived service quality has the potential to influence membership renewal decisions. Previous studies conducted in Ghana have also highlighted service quality as a key factor in ensuring an optimal NHIS subscriber base. For example, Boateng and Awunyor-Vitor pointed out that 58% of respondents in their study cited poor quality as a reason for not renewing their membership [16]. This is based on the evidence that customer satisfaction and behavioral intentions are influenced by the perceived quality of services they receive [48]. The findings of our study are also consistent with those of Kumi-Kyereme et al., who identified poor service quality as a major barrier to NHIS membership [14].

Studies from other developing countries further highlight the effect of health service quality on social health insurance programs in low-resource settings, as discussed in this study. For instance, in Guinea, poor quality of care provided to mutual health insurance members at health centers was the primary reason for the lack of interest in the program [47]. In Ethiopia, the accessibility of medical supplies and the waiting time to consult a doctor were found to have a significant influence on health insurance uptake decisions [45]. Elsewhere in Nepal, a study demonstrated that unfriendly behavior of health workers, insufficiency of drugs and other essential supplies, and long waiting times for health services were significant factors that affect health insurance status [51]. These findings all underscore the interrelationship between multidimensional service quality and health insurance membership decisions as illustrated in this study.

The present study found that the multidimensional perceived quality of health services under Ghana’s NHIS can offer a potential explanation for the observed non-renewal. To secure an optimal risk pool and maximize enrollee retention, our findings highlight the importance of close collaboration among relevant state institutions, notably the National Health Insurance Authority (NHIA), the Ministry of Health, and the Ghana Health Service, to design and enforce service quality standards for NHIS-accredited facilities. More critically, the perspectives of the current and potential NHIS members in the informal sector should be incorporated in setting the service quality standards. In addition, the government of Ghana and other developing countries with similar health insurance schemes should invest in expanding healthcare infrastructure and human resources to accommodate the growing needs of the insured population.

### Limitations

Despite meaningful findings, some limitations remain. First, this study employed convenience sampling, drawing those who were available and willing to participate rather than using probability sampling. The study sample may therefore not be statistically representative of NHIS members in the study location. Specifically, there was a challenge in obtaining a reliable, up-to-date list of NHIS members with addresses to help create a sample frame. Building one would have taken more time and resources beyond the budget of this study. However, key steps were taken to recruit broader and diverse respondents as much as possible to ensure the results were trustworthy and policy-relevant. So despite the limitations of convenience sampling, the study offers valuable insights into how service quality may influence NHIS membership renewal and long-term sustainability of the scheme in Ghana. Future studies can highlight potential variations in the perceived health service quality by applying probability sampling with diverse occupation categories in the informal sector.

Again, the study focused only on the informal sector and did not include other categories of NHIS membership. The study therefore missed the opportunity to explore the extent to which perceived quality may differ across various membership groups. Future studies should consider recruiting diverse membership types, including the exempt, to examine how membership type may influence quality perceptions under NHIS in Ghana. Also, missing values and inapplicable questions reduced the sample size, thus limiting the possibility of advanced analysis such as the effect of perceived service quality mediated through sociodemographic characteristics. Future research can address these limitations to further investigate the role of service quality in NHIS retention dynamics with a broader population.

There was also the potential for post-decision rationalization. That is, those who dropped out may have retrospectively rated service quality more negatively than they perceived, to justify their decision not to renew. Lastly, this study does not incorporate clinical outcomes as part of the measurement framework by focusing on the client perspective. Although this study does not support causal inferences due to its cross-sectional design, a longitudinal study that incorporates clinical outcomes can advance the understanding of the complex relationship between health service quality and NHIS membership decisions. Despite these limitations, this study draws attention to the multidimensionality of perceived service quality from the community perspective and how that may influence membership of public health insurance programs in emerging economies.

## Conclusions

Upon reaching the two-decade milestone of the NHIS in Ghana, this study examined the multidimensional service quality from the perspective of clients and demonstrated how it can be associated with their membership renewal decisions. Focusing on the informal sector workers as a critical subgroup of the NHIS subscriber base, this study underscores the potential role of service quality from the client perspective in facilitating membership retention. The findings suggest that perceived service quality across three dimensions, particularly in terms of resource availability, patient flow, and relational quality, can be pivotal in sustaining enrollment. With the increasing attention to client-oriented perspective in health service quality measurement, the empirical evidence from this study provides insights into the policy reforms for health service quality in Ghana as well as in other low-resource settings. Ultimately, this research can inform future policy on how better service quality from the client perspectives can enhance membership retention in national health insurance schemes.

## Supplementary Material

Supplementary File 2 Appendix.docx

Supplementary File 1.docx

STROBE final revised.doc

## Data Availability

The data that support the findings of this study are available from the authors upon request.
