# Supplementary material for: Two decades of Ghana’s National Health Insurance Scheme: perceived service quality and membership renewal among informal sector workers
Source: Glob Health Action. 2026 Nov 3;19(1):2755137. doi: 10.1080/16549716.2026.2755137 (PMC13757927; doi:10.1080/16549716.2026.2755137)
Supplement: Supplementary file 1 [file ZGHA_A_2755137_SM0060.docx]

**SECTION A: Respondent's Socio-demographic Information**

**A1**: Sex of respondent [0] Female [1] Male

**A2**: Age of respondent [1] 18-29 [2] 30-39 [3] 40-49 [4] 50-59 [5] 60-69

**A3**: Level of education of respondent [1] No Formal Education [2] Basic Education [3] Senior Secondary/Tech/Voc school [4] Tertiary

**A4**: Religion of respondent [1] African Traditional Religion [2] Christianity [3] Islam [4] Others

**A5:** Region of the respondent [1] Northern Region [2] Greater Accra Region

**A6:** Residence of the respondent [1] Rural [2] Urban

**A7**: Marital status of the respondent [1] Single [2] Married [3] Widowed/divorced [4] Not married but live with a partner

**A8:** The household size of the respondent [0] Less than 5 [1] 5 and More

**A9**: Are you a parent of child (ren) under 18 years old? [1] No [2] Yes

**A10** Primary occupation of the respondent [1] Food/street vendor [2] Artisan [3] Laborer [4] Trader/business person [5] Commercial motor and vehicle driver [6] Farmer [7] Other

**A11** Approximate monthly income level of the respondent (In GHC) [1] Less than 100 [2] 100–399 [3] 400–699, [4] 700–999 [5] 1000+

**SECTION B: Awareness, Source of Information and Perceptions About Health Insurance**

**B12:** Are you aware of the national health insurance scheme? (0) Unaware (1) Aware

**B13:** From whom do you get information about NHIS in your community? Please select the most important/frequent source.

| Relatives, friends, neighbors |  |
| --- | --- |
| Religious leaders (Church/Mosque) |  |
| Groups and associations |  |
| Co-workers (Workplace) |  |
| Community leaders |  |
| (Local) Government officer |  |
| Media (Newspaper, TV/Radio, Mobile phone, Poster, Billboard) |  |

**B14** How much information do you have about NHIS?

[1] Not sufficient at all [2] Not sufficient [3] Somewhat Sufficient [4] Sufficient [5] Very sufficient

**B15** How much information do you have about NHIS premium level?

[1] Not sufficient at all [2] Not sufficient [3] Somewhat Sufficient [4] Sufficient [5] Very sufficient

**B16:** How much information do you have about NHIS exemption policy?

[1] Not sufficient at all [2] Not sufficient [3] Somewhat Sufficient [4] Sufficient [5] Very sufficient

***Note:The exemption policy is the policy that allows for some segments of the population not to pay yearly premiums***

**B17:** How much information do you have about NHIS registration processes?

[1] Not sufficient at all [2] Not sufficient [3] Somewhat Sufficient [4] Sufficient [5] Very sufficient

**B18** How much information do you have about NHIS renewal processes?

[1] Not sufficient at all [2] Not sufficient [3] Somewhat Sufficient [4] Sufficient [5] Very sufficient

**B19** How much information do you have about medicines that are covered under the NHIS?

[1] Not sufficient at all [2] Not sufficient [3] Somewhat Sufficient [4] Sufficient [5] Very sufficient

**SECTION C: Perceived Quality of NHIS Operations/Services**

**C20 Please indicate your level of satisfaction of the following aspect of the NHIS**

| **Statement** | **Measurement** |
| --- | --- |
| **A** How satisfied are you with the services provided by the district NHIS office? | very satisfied **5\|4 \|3 \|2 \|1** very dissatisfied |
| **B** How satisfied are you with the information provided by the district NHIS office? | very satisfied **5\|4 \|3 \|2 \|1** very dissatisfied |
| **C** How satisfied are you about the complaint handling by the NHIS? | very satisfied **5\|4 \|3 \|2 \|1** very dissatisfied |
| **D** How satisfied are you about the benefit you derive from the NHIS? | very satisfied **5\|4 \|3 \|2 \|1** very dissatisfied |
| **E** How satisfied are you about the medicine list under the NHIS? | very satisfied **5\|4 \|3 \|2 \|1** very dissatisfied |
| **G** How satisfied are you with the distance from the NHIS office to your house? | very satisfied **5\|4 \|3 \|2 \|1** very dissatisfied |
| **H** How satisfied are you with the NHIS registration and renewal process? | very satisfied **5\|4 \|3 \|2 \|1** very dissatisfied |

**SECTION D: Perceived Trust in NHIS**

I want to kindly ask you some questions about your perception of Ghana's national health insurance scheme. Specifically, I will read out some trust-related statements to you, and you will please indicate to me how much you agree or disagree with each statement. The responses include; Strongly Agree (5), Agree (4), Neutral (3), Disagree (2), and Strongly Disagree (1).

**D21**

| **Statement** | **Measurement** |
| --- | --- |
| **A** The premium money is used for its own purposes (e.g. providing treatment) only. | Strongly agree **5\|4 \|3 \|2 \|1** Strongly disagree |
| **B** You believe the national health insurance will pay for everything it is supposed to, even costly treatments | Strongly agree **5\|4 \|3 \|2 \|1** Strongly disagree |
| **C** The NHIS is trustworthy. | Strongly agree **5\|4 \|3 \|2 \|1** Strongly disagree |

**SECTION E: Health Status, NHIS membership and Healthcare Utilization**

**E22** How will you rate your present health condition [1] Very poor [2] Poor [3] Fair [4] Good

[5] Very good

**E23:** In the past 12 months, have you fallen sick and visited the hospital as an outpatient [0] No [1] Yes

**E24** In the past 12 months, have you fallen sick and stayed in the hospital for at least one night? [0] No [1] Yes

**E25** Please indicate the type of health facility you visited most frequently in the past 12 months. [1] CHPS compound [2] Health center [3] Polyclinic hospital [4] Teaching hospital [5] Pharmacy

**E26** The last time you visited a NHIS health provider did you have to pay (out of pocket) for the following

|  | No | Yes |
| --- | --- | --- |
| Consultation |  |  |
| Tests or laboratory services |  |  |
| Drugs |  |  |

**E27** Are you enrolled or ever enrolled in the national health insurance scheme?

[1] Renewed from the previous membership

[2] Previously enrolled but now dropped

[3] Never Enrolled

**E28** If currently enrolled, are you willing to renew your membership on the next expiry date? [0] Not willing; [1] willing to renew

**E29 Reasons for enrolling (Please select the most important reason to you)**

[1] NHIS provides financial protection against illness.

[2] The community opinion leader asked me to join.

[3] Relatives or close friends asked me to join.

[4] The government recommended joining.

[5] The NHIS membership makes it easier for health workers to get my information.

**E30** **Reasons for never enrolling or drop-out (Please select the most important reason to you)**

| Cannot afford premium or renewal payment |  |
| --- | --- |
| I am mostly healthy do not need to insure |  |
| Have private health insurance |  |
| No confidence in the scheme |  |
| Poor health service quality |  |
| Registration point too far |  |
| Health facility too far |  |
| Drugs should be purchased outside facility anyway |  |
| Longer Waiting times at NHIS |  |
| Inconvenience in premium payment |  |
| Other |  |

**SECTION F: Perceived Healthcare Quality Under NHIS**

I would now like to ask you some questions about the following characteristics of healthcare services at NHIS-approved health facility that you visited the last time. Each item will be adequately explained before you proceed to respond to the questions that follow.

| **1** | **Waiting time before seeing the doctor**: The time that you waited in the hospital before seeing the doctor. |
| --- | --- |
| **2** | **Availability of Medical and laboratory equipment**: the availability of equipment for all prescribed laboratory test in the health facility laboratory |
| **3** | **Waiting time for laboratory tests or examinations:** the time that you waited to have the required tests or examinations conducted (e.g. blood tests, scans and x-rays). |
| **4** | **Availability of drugs:** the availability of the prescribed medicine in the NHIS-accredited health facility |
| **5** | **Staff attitude/disposition:** the professionalism of the workers, including those in the reception area, nurses and physicians (health personnel). |
| **6** | **Doctor-patient relationship:** the time spent with the doctor to discuss problems and state of health, and the receipt of sufficient information about how to improve your health. etc |

*(****Prompt****:* ***Confirm if the respondent has understood what these characteristics mean and provide further clarifications if needed)***

**Waiting Time to See a Doctor**

**F31** Based on the last visit, how long did you wait before seeing the doctor?

[1] Less than 30 minutes [2] Between 30 to less than 60 minutes [3] Between 60 to less than 90 minutes [4] Between 90 to less than 120 minutes [5] 120 minutes and above

**F32** How do you consider this length of waiting time before seeing the doctor? [1] Very long [2] Long [3] Acceptable [4] Not long [5] Not long at all

**Waiting time for Laboratory test**

**F33** Based on the last visit, did you have any laboratory tests or investigations (for example blood tests, scans or X-rays)? [0] No [1] Yes

**F34** Based on the last visit, how long did you wait in order to have the requested laboratory tests or examinations conducted? [1] Less than 30 minutes [2] Between 30 to less than 60 minutes [3] Between 60 to less than 90 minutes [4] Between 90 to less than 120 minutes [5] 120 minutes and above

**F35** How do you consider this length of waiting time in order to have the requested laboratory tests or examinations conducted at the public hospital? [1] Very long [2] Long [3] Acceptable [4] Not long [5] Not long at all.

**Availability of Drugs and Laboratory Equipment**

**F36** At the last visit, did the doctor at the NHIS-accredited health facility prescribe any medicines? [0] No [1] Yes

**F37** What was the availability of the drugs prescribed by the doctor at the NHIS-accredited health facility pharmacy? [1] All of them were available [2] Only some of them were available [3] None of them were available

**F38** At the last visit, did the doctor at the public hospital recommend any laboratory test to you? [0] No [1] Yes

**F39** What was the availability of these test at the NHIS-accredited health facility [1] All of them were available [2] Only some of them were available [3] None of them were available.

**Disposition of hospital staff** (*including the courtesy, compassion and professionalism of the staff in reception, nurses)*

**F40** Based on the last visit, how would you rate the disposition of the hospital staff towards patients? [1] Not Professional at all [2] Not professional [3] Somewhat professional [4] Professional [5] Very professional

**Doctor-patient Relationship**

**F41** Based on the last visit, indicate your degree of agreement or disagreement with each of the following statements?

| **Statement** | **Measurement** |
| --- | --- |
| 1. Physician spent sufficient time with me. | Strongly agree **5\|4 \|3 \|2 \|1** Strongly disagree |
| 2. Physician undertook comprehensive examinations for my illness. | Strongly agree **5\|4 \|3 \|2 \|1** Strongly disagree |
| 3. Physician clearly explained my health problem to me in simple terms | Strongly agree **5\|4 \|3 \|2 \|1** Strongly disagree |
| 4. Physician provided me with sufficient information on what I should do to improve my health. | Strongly agree **5\|4 \|3 \|2 \|1** Strongly disagree |

**F42** In general, how would you express the overall level of your satisfaction with the current quality of healthcare services at public hospitals? [1] Not satisfied [2] satisfied

**F43**The health service I receive the at hospital with my health insurance card is worthy of the money that I have spent as health insurance cost [1] Strongly disagree [2] Disagree [3] Neither disagree or Agree [4] Agree [5] Strongly Agree

**SECTION G: Health Risk Attitude**

**G44** Please indicate how much you Agree or Disagree about the following.

| **Statement** | **Measurement** |
| --- | --- |
| I think I take good care of my body | Strongly agree **5\|4 \|3 \|2 \|1** Strongly disagree |
| It is vital to me that I organize my life  so that I can later enjoy good health | Strongly agree **5\|4 \|3 \|2 \|1** Strongly disagree |
| If it concerns my health, then I  see myself as someone who avoids risks | Strongly agree **5\|4 \|3 \|2 \|1** Strongly disagree |
| My health means everything to me | Strongly agree **5\|4 \|3 \|2 \|1** Strongly disagree |
| It is safety first, where my health is concerned | Strongly agree **5\|4 \|3 \|2 \|1** Strongly disagree |
| I am prepared to give up a lot to enjoy good health now and in the future | Strongly agree **5\|4 \|3 \|2 \|1** Strongly disagree |
