# Supplementary material for: Two decades of Ghana’s National Health Insurance Scheme: perceived service quality and membership renewal among informal sector workers
Source: Glob Health Action. 2026 Nov 3;19(1):2755137. doi: 10.1080/16549716.2026.2755137 (PMC13757927; doi:10.1080/16549716.2026.2755137)
Supplement: Supplementary file 2 [file ZGHA_A_2755137_SM0061.docx]

**Appendix 1: Test for multicollinearity**

| **Independent variables** | **Collinearity statistics** | |
| --- | --- | --- |
|  | **Tolerance** | **VIF** |
| Gender (ref: male) | 0.90 | 1.11 |
| Age (ref: ≤ 40) | 0.88 | 1.14 |
| Marital status (ref: single) | 0.89 | 1.12 |
| Education (ref: no formal education) | 0.89 | 1.13 |
| Region (ref: Northern) | 0.93 | 1.07 |
| Residence (ref: peri-urban) | 0.86 | 1.17 |
| Availability of medications  (ref: none was available) | 0.85 | 1.18 |
| Availability of diagnostic services (ref: none was available) | 0.93 | 1.07 |
| Waiting time for consultation (ref: long) | 0.76 | 1.31 |
| Waiting time for diagnostics (ref: long) | 0.86 | 1.17 |
| Staff disposition towards NHIS users (ref: not professional) | 0.89 | 1.12 |
| Doctor-patient relationship | 0.86 | 1.17 |

**Appendix 2: Box-Tidwell nonlinearity test**

| **NHIS renewal** | **Coeff.** | **Std. err.** | **Z** | **P-value** | **CI: 95%** |
| --- | --- | --- | --- | --- | --- |
| DPR, estimated power transformation term | .766 | 1.01 | 0.76 | 0.447 | -1.21, 2.74 |
| DRP*ln(DPR), nonlinearity term | .003 | .49 | 0.01 | 0.995 | -.96, .96 |
| _cons | .99 | .14 | 7.13 | 0.000 | .72, 1.26 |
| DPR (linear term) | 1.19 | .16 | 7.11 Nonlin. dev. 0.071 (P = **0.789**) | | |
| Estimated power parameter | 1.20 | .63 |  |  |  |

DPR: doctor-patient relationship
