# Supplementary material for: Two decades of Ghana’s National Health Insurance Scheme: perceived service quality and membership renewal among informal sector workers
Source: Glob Health Action. 2026 Nov 3;19(1):2755137. doi: 10.1080/16549716.2026.2755137 (PMC13757927; doi:10.1080/16549716.2026.2755137)
Supplement: Supplementary file 3 [file ZGHA_A_2755137_SM0059.doc]

STROBE Statement—Checklist of items that should be included in reports of ***cross-sectional studies***

|  | Item No | Page/Location | | Recommendation |
| --- | --- | --- | --- | --- |
| **Title and abstract** | 1 | 1/Lines 29-32 | | (*a*) Indicate the study’s design with a commonly used term in the title or the abstract |
|  |  | 1/Lines 33-44 | | (*b*) Provide in the abstract an informative and balanced summary of what was done and what was found |
|  | | | Introduction | |
| Background/rationale | 2 | 3-4/Lines 77-98 | | Explain the scientific background and rationale for the investigation being reported |
| Objectives | 3 | 6/Lines 135-142 | | State specific objectives, including any prespecified hypotheses |
|  | | | Methods | |
| Study design | 4 | 6/Lines 146-147 | | Present key elements of study design early in the paper |
| Setting | 5 | 6/Lines 147-154 | | Describe the setting, locations, and relevant dates, including periods of recruitment, exposure, follow-up, and data collection |
| Participants | 6 | 7-8/Lines 175-181 | | (*a*) Give the eligibility criteria, and the sources and methods of selection of participants |
| Variables | 7 | 8, 10/Lines 187-189, 236-239 | | Clearly define all outcomes, exposures, predictors, potential confounders, and effect modifiers. Give diagnostic criteria, if applicable |
| Data sources/ measurement | 8 | 9-10/Lines 223-239 | | For each variable of interest, give sources of data and details of methods of assessment (measurement). Describe comparability of assessment methods if there is more than one group |
| Bias | 9 | 7/Lines 155-158 | | Describe any efforts to address potential sources of bias |
| Study size | 10 | 7/Lines 167-171 | | Explain how the study size was arrived at |
| Quantitative variables | 11 | 11/Lines 259-267 | | Explain how quantitative variables were handled in the analyses. If applicable, describe which groupings were chosen and why |
| Statistical methods | 12 | 11-12/Lines 253-284 | | (*a*) Describe all statistical methods, including those used to control for confounding |
|  |  | 11-12/Lines 273-279 | | (*b*) Describe any methods used to examine subgroups and interactions |
|  |  | 13/Lines 281-284 | | (*c*) Explain how missing data were addressed |
|  |  | NA | | (*d*) If applicable, describe analytical methods taking account of sampling strategy |
|  |  | 18/Lines 379-382 | | (*e*) Describe any sensitivity analyses |
|  | | | Results | |
| Participants | 13* | 12/Lines 288-294 | | (a) Report numbers of individuals at each stage of study—eg numbers potentially eligible, examined for eligibility, confirmed eligible, included in the study, completing follow-up, and analysed |
|  |  | 17/Lines 356-358 | | (b) Give reasons for non-participation at each stage |
|  |  | 13/Figure 2 | | (c) Consider use of a flow diagram |
| Descriptive data | 14* | 13-14/298-305 | | (a) Give characteristics of study participants (eg demographic, clinical, social) and information on exposures and potential confounders |
|  |  | 14/317-320 | | (b) Indicate number of participants with missing data for each variable of interest |
| Outcome data | 15* | 12/Lines 288-293 | | Report numbers of outcome events or summary measures |
| Main results | 16 | 16, 16-17/Lines 342-345 (Appendix 2), 358-382 (Table 3) | | (*a*) Give unadjusted estimates and, if applicable, confounder-adjusted estimates and their precision (eg, 95% confidence interval). Make clear which confounders were adjusted for and why they were included |
|  |  | NA (no categorization of continuous variable) | | (*b*) Report category boundaries when continuous variables were categorized |
|  |  | NA | | (*c*) If relevant, consider translating estimates of relative risk into absolute risk for a meaningful time period |
| Other analyses | 17 | 18/Lines 379-382 (Appendix 3) | | Report other analyses done—eg analyses of subgroups and interactions, and sensitivity analyses |
|  | | | Discussion | |
| Key results | 18 | 20/Lines 409-413 | | Summarise key results with reference to study objectives |
| Limitations | 19 | 22-23/Lines 474-495 | | Discuss limitations of the study, taking into account sources of potential bias or imprecision. Discuss both direction and magnitude of any potential bias |
| Interpretation | 20 | 20-21/Lines 418-449 | | Give a cautious overall interpretation of results considering objectives, limitations, multiplicity of analyses, results from similar studies, and other relevant evidence |
| Generalisability | 21 | 22/Lines 470-472 | | Discuss the generalisability (external validity) of the study results |
|  | | | Other information | |
| Funding | 22 | 25/Lines 534-538 | | Give the source of funding and the role of the funders for the present study and, if applicable, for the original study on which the present article is based |
